# The Viable Mitral Annular Dynamics and Left Ventricular Function after Mitral Valve Repair by Biological Rings

**Published:** 2012-12-15

**Authors:** Farideh Roshanali, Ali Vedadian, Saeed Shoar, Saleh Sandoughdaran, Mohammad Naderan, Mohammad Hossein Mandegar

**Affiliations:** 1 Department of Cardiology, Day General Hospital, Tehran, IR Iran; 2 Department of Cardiac Surgery, Tehran University of Medical Sciences, Shariati Hospital, Tehran, IR Iran; 3 School of Medicine, Tehran University of Medical Sciences, Tehran, IR Iran; 4 Department of Surgery, Shariati Hospital, Tehran University of Medical Sciences, Tehran, IR Iran

**Keywords:** Mitral Valve, Valvuloplasty, Mitral Valve Regurgitation

## Abstract

**Objective:**

Considering the importance of annular dynamics in the valvular and ventricular function, we sought to evaluate the effects of treated pericardial annuloplasty rings on mitral annular dynamics and left-ventricular (LV) function after mitral valve repair. The results were compared with the mitral annular dynamics and LV function in patients with rigid and flexible rings and also in those without any heart problems.

**Materials and Methods:**

One hundred and thirty-six consecutive patients with a myxomatous mitral valve and severe regurgitation were prospectively enrolled in this observational cohort study. The patients underwent comparable surgical mitral valve reconstruction; of these 100 received autologous pericardium rings (Group I), 20 were given flexible prosthetic rings (Group II), and 16 received rigid rings (Group III). Other repair modalities were also performed, depending on the involved segments. The patients were compared with 100 normal subjects in whom an evaluation of the coronary artery was not indicative of valvular or myocardial abnormalities (Group IV). At follow-up, LV systolic indices were assessed via two-dimensional echocardiography at rest and during dobutamine stress echocardiography. Mitral annular motion was examined through mitral annulus systolic excursion (MASE). Peak transmitral flow velocities (TMFV) and mitral valve area (MVA) were also evaluated by means of continuous-wave Doppler.

**Results:**

A postoperative echocardiographic study showed significant mitral regurgitation (>=2+) in one patient in Group I, one patient in Group II, and none in Group III. None of the patients died. There was a noteworthy increase in TMFV with stress in all the groups, the increase being more considerable in the prosthetic ring groups (Group I from 1.10 ± 0.08 to 1.36 ± 0.13 m/s, Group II from 1.30 ± 0.11 to 1.59 ± 0.19 m/s, Group III from 1.33 ± 0.09 to 1.69 ± 0.21 m/s, and Group IV from 1.08 ± 0.08 to 1.21 ± 0.12 m/s). Recruitment of LVEF reserve during stress was observed in the pericardial ring and normal groups (Group I from 54.6±6.2 to 64.6±7.3%, P<0.005; and Group IV from 55.3 ± 5.7 to 66 ± 6.2%, P<0.05), but no significant changes were detected in the prosthetic ring groups (Group II from 50.4 ± 5 to 55.0 ± 5.1, and Group III from 51.1 ± 6.6 to 53.8 ± 4.7). There was a significant MASE increase in both of the studied longitudinal segments at rest and during stress in Groups I and IV compared with the prosthetic ring groups. There was no calcification of the pericardial rings.

**Conclusions:**

The use of treated autologous pericardium rings for mitral valve annuloplasty yields excellent mitral annular dynamics, preserves LV function during stress conditions, and leaves no echocardiographic signs of degeneration.

## 1. Background

Valve repair has recently become the treatment of choice for mitral regurgitation(1-4), partly owing to the interactive contribution of the mitral annulus to the physiology of the valvular apparatus throughout the cardiac cycle(5, 6) . The contraction and relaxation of the muscle bundles that posteriorly surround the mural portion of the mitral annulus continuously change the size and topology of the annulus during systole and diastole. There is evidence that left-ventricular (LV) function is influenced by the role played by annular dynamics in the valve-ventricle interaction. Such annular dynamics should, therefore, be preserved in mitral valve repair (MVR) techniques by averting geometrical deformity and excessive fixation in the course of annuloplasty(7-13) .

The effects of repair with rigid and flexible rings on annular motion and LV function have been compared in experimental and clinical settings, producing controversial results(14-22) . Still, many seem to opt for flexible annuloplasty devices for their less restrictive effects on annular dynamics and better preservation of both valve-ventricle interaction and LV function (15, 16, 20-22) .The autologous pericardium is an additional option for the annuloplasty procedure,(23, 24) with numerous clinical studies into the use of such biological tissues demonstrating their excellent long-term efficacy(25, 26) . There have been only a few early or late comparative evaluations thus far between prosthetic and pericardial rings in MVR, but they all confirm the superiority of autologous rings over prosthetic ones(27-29) .

This study was performed to evaluate the effects of treated pericardial annuloplasty rings on mitral annular dynamics and LV function after MVR in a selected group of patients.

## 2. Materials and methods

### 2.1. Patients

This prospective observational cohort study recruited 136 consecutive patients who underwent MVR for mitral valve regurgitation secondary to prolapse of the leaflets at Day General Hospital between May 1999 and November 2002. The inclusion criteria were: 1) presence of pure mitral insufficiency, 2) absence of calcified mitral annulus, 3) absence of rheumatic etiology, 4) absence of active endocarditis, 5) preserved LV function, and 6) absence of coronary artery disease.

All the 136 patients, who had severe preoperative mitral regurgitation secondary to myxomatous degeneration, underwent surgical correction of the valvular inufficiency, including quadrangular resection, chorda transfer, artificial chorda, commissuroplasty, or other kinds of repair depending on the valve involvement in conjunction with the annuloplasty procedure. One hundred patients received autologous pericardium rings (Group I), 20 received flexible prosthetic rings (Group II), and 16 were given classic rigid rings (Group III). Another 100 people in whom an evaluation of the coronary artery had shown no valvular or myocardial abnormalities were selected as Group IV.

### 2.2. Surgical technique

All the surgical operations were performed through a conventional median sternotomy. Moderate hypothermic (28-30˚c) cardiopulmonary bypass was instituted using bicaval and ascending aortic cannulation. Myocardial protection was achieved with antegrade/retrograde cold blood cardioplegia and topic cooling. After aortic cross-clamping, mitral valve was exposed through an extended transseptal incision. Chordal replacement with Gore-Tex sutures, chordal transfer, quadrangular resection of the posterior leaflet, and sliding plasty were all used to correct leaflet prolapse depending on the type of the prolapsing portion of the mitral leaflet with ruptured or elongated chordae.

In Group I patients, a rectangular patch of autologous pericardium (about 3x7 cm^2^) was harvested, which was not subsequently fixed with glutaraldehyde-buffered solution. Prior to implantation, a pericardial ring (8mm wide, C-shaped strip) was cut from the patch. The length of the pericardial ring was proportioned to the free-edge extension of the anterior leaflet. The pericardial ring was folded over, so that the internal pericardial surface faced inwards; subsequently, it was fixed with mattress sutures along the posterior annulus, just beyond the anatomic commissures. Mitral- leaflet continuity was restored by suturing the opposing edges with 5-0 prolene single-mattress stitches, followed by a single-running suture.

In the prosthetic ring groups, the prosthetic rings were measured with a Carpentier-Edwards sizer based on the anterior leaflet surface extension. The rings were implanted with 2-0 prolene.

After MVR, the LV was forcefully filled with saline solution to test the valve competence. Intraoperative transesophageal echocardiography as well as postoperative echocardiographic assessments was carried out routinely before hospital discharge. All the patients were treated with anticoagulant therapy for the first three postoperative months.

### 2.3. Echocardiographic study

Dobutamine stress echocardiography was performed once for all the patients at the last follow-up visit. Real-time phased array echo-Doppler recordings were obtained at rest and during dobutamine infusion with a GE Medical System, Vivid 7, Horton, Norway. Digital images were stored on a CD for subsequent analyses. The LV diameters and volumes (area-length method) were determined from two-dimensional parasternal long-axis view and from apical four-chamber view, respectively. Calculations of LV fractional shortening and left-ventricular ejection fraction (LVEF) were then derived from the diameters and volumes, respectively. Continuous-wave Doppler was used to obtain transmitral flow velocity curves; both mean and peak velocities were taken into consideration. An assessment of the longitudinal atrio-ventricular plane displacement was made, with 2-dimensionally-guided M-mode from the apical 4-chamber view for the septal (S) and lateral (L) segments, to elucidate regional mitral annulus systolic excursion (MASE) in relation to LV segmental mechanics. Starting from Q wave on ECG tracing, the maximal excursion of M-mode tracing during systole was considered. The measurements of all the echo-Doppler examinations were performed at rest and during peak stress of dobutamine infusion.

Resting transthoracic echocardiography was followed by dobutamine stress echocardiography. Dobutamine infusion was commenced with 10 μg/kg/min, which was subsequently increased to 20, 30, and 40μg/kg body weight per minute in 3-minute stages. At each stage, blood pressure and heart rate were measured. The end points of the test were obtained by achieving maximal heart rate (85% of 220-age in men and 210-age in women), significant ventricular and supraventricular arrhythmia, significant bradyarrythmia, increased blood pressure of more than 240/120mmHg, drop of 20mmHg in systolic blood pressure by compared with the baseline value, new wall-motion abnormality, ST depression of 2mm or more, and severe angina. The mean value of the three measurements was considered for each variable.

### 2.4. Statistical analysis

All the results are expressed as mean ± standard deviation. The comparisons between categorical data and continuous variables were made by Chi-square test and independent sample t-test respectively. A non-parametric Mann–Whitney test was utilized when the variable distribution was found to be normal by the Kolmogorov–Smirnov test. A P-value of <0.05 was considered statistically significant.

## 3. Results

Duration of follow-up was 24-65 months, with a mean of 58±7 months. There was no experience of major complications. Re-operation was not needed for any of the patients to avoid the recurrence of hemodynamically-significant mitral regurgitation during the follow-up.

Heart rate and systolic blood pressure values at rest were almost similar in all the groups. An echocardiographic evaluation at rest did not show any significant difference in terms of LV diameters, LV fractional shortening, LVEF, and mean and peak transmitral flow velocities (TMFV). Maximal heart rate was achieved by dobutamine infusion in all the groups. No mitral regurgitation was induced by the stress conditions in any patient.

A postoperative echocardiographic study revealed significant mitral regurgitation (>=2+) in one patient in Group I, one patient in Group II, and none in Group III, with no reported mortality.

There was a significant increase in TMFV with stress in all the groups, the increase being more significant in the prosthetic ring groups (Group I from 1.10 ± 0.08 to 1.36 ± 0.13 m/s, Group II from 1.30 ± 0.11 to 1.59 ± 0.19 m/s, Group III from 1.33 ± 0.09 to 1.69 ± 0.21, and Group IV from 1.08 ± 0.08 to 1.21 ± 0.12).

Recruitment of LVEF reserve during stress was observed in the pericardial ring and normal groups (Group I from 54.6±6.2 to 64.6±7.3%, P<0.005; and Group IV from 55.3 ± 5.7 to 66 ± 6.2%, P<0.05), but no significant changes were seen in the prosthetic ring groups (Group II from 50.4 ± 5 to 55.0 ± 5.1, and Group III from 51.1 ± 6.6 to 53.8 ± 4.7).

There was a significant increase in MASE at both the studied longitudinal segments at rest and during stress in Groups I and IV compared with the prosthetic ring groups. Pericardial degeneration, fibrous overgrowth, or calcium depots were not observed in any of the patients. Symptomatic improvement was noted in most of the patients. The New York Heart Association (NYHA) functional class (1.2±0.6) at follow-up was significantly better than that of pre-operation (2.5±0.5), with a mean improvement of 1.3 NYHA class (CL90%: 1.2–1.4 NYHA class) (P<0.0001). Mitral valve area was 2.8±0.4 cm2 in Group I, 2.0 cm2 in Group II, and 2.3 cm2 in Group III. At follow-up, no patients exhibited SAM with significant LVOT obstruction.

All the variables were correlated with follow-up in each group, and no statistical relationship was found between the hemodynamic changes and the timing of postoperative evaluation.

**Table1.** Characteristics and Primary Features of Study Patients

Variable	pericardial ring Group I	Flexible ring Group II	Rigid ring Group III	Normal Group IV	*P* Value	Difference between groups
Sex (M/F)	56/44	12/8	9/7	46/54	0.438	---
Age (Mean ± SD)	50.7 ± 12.1	49.9 ± 8.8	49.3 ± 6.4	50.9 ±11.9	0.944	---
HR – Rest (Mean ± SD)	71.9 ± 11.0	78.1 ± 15.0	83.6 ± 12.4	76.0 ±12.6	0.001	Group I vs. others
HR – Peak stress echo (Mean ± SD)	147.8 ± 13.8	146.7 ± 8.7	148.4 ± 9.6	145.8 ±9.7	0.647	---
Increase in HR at stress echo (Mean ± SD)	75.9 ± 17.9	68.6 ±14.9	64.8 ±14.4	69.8 ±15.4	0.012	Group I vs. Groups III and IV
SBP – Rest (Mean ± SD)	121.0 ± 12.9	122.8 ± 9.4	118.1 ± 11.1	120.5 ±11.9	0.709	---
SBP – Peak stress echo (Mean ± SD)	162.4 ± 20.8	166.5 ± 22.5	164.4 ± 25.3	164.4 ±23.3	0.858	---
Increase in SBP at stress echo (Mean ± SD)	41.4 ± 21.5	43.8 ± 18.8	46.3 ± 21.9	43.9 ± 22.8	0.782	---
EF – Rest (Mean ± SD)	54.6 ±6.2	50.4 ± 5.0	51.1 ± 6.6	55.3 ± 5.7	0.001	Groups II & IV vs. Groups II & III
EF – Peak stress echo (Mean ± SD)	64.6 ± 7.3	55.0 ± 5.1	53.8 ± 4.7	66.0 ± 6.2	< 0.0001	Groups II & IV vs. Groups II & III
Increase in EF at stress echo (Mean ± SD)	10.0 ± 4.7	4.6 ± 4.3	2.6 ± 3.6	10.7 ± 4.6	< 0.0001	Groups II & IV vs. Groups II & III
TMFV – Rest (Mean ± SD)	1.10 ± 0.08	1.30 ± 0.11	1.33 ± 0.09	1.08 ± 0.08	< 0.0001	Group II equal Group III and others different
TMFV – Peak stress echo (Mean ± SD)	1.36 ± 0.13	1.59 ± 0.19	1.69 ± 0.21	1.21 ± 0.12	< 0.0001	All groups different
Increase in TMFV at stress echo (Mean ± SD)	0.26 ± 0.12	0.29 ± 0.24	0.36 ± 0.22	0.13 ± 0.13	< 0.0001	Group IV vs. others and Group I vs. Group III

**Table2.** Comparison of MASE between the 4 Groups

	Pericardial ring	Flexible ring	Rigid ring	Normal	*P* Value	Difference between groups
MASE_L – Rest (Mean ± SD)	12.9 ± 1.3	9.8 ± 1.7	9.0 ± 1.8	13.1 ± 1.7	< 0.0001	Groups II & IV vs. Groups II & III
MASE_L – Peak stress echo (Mean ± SD)	20.1 ± 2.2	13.3 ± 2.5	11.7 ± 2.2	20.2 ± 2.3	< 0.0001	Group I equal Group IV and others different
Increase in MASE_L at stress echo (Mean ± SD)	7.2 ± 2.1	3.5 ± 2.5	2.7 ± 1.7	7.1 ± 2.8	< 0.0001	Groups II & IV vs. Groups II & III
MASE_S – Rest (Mean ± SD)	12.4 ± 1.5	9.6 ± 1.0	9.2 ± 1.6	13.1 ± 1.8	< 0.0001	Group II equal Group III and others different
MASE_S –Peak stress echo (Mean ± SD)	19.4 ± 2.3	12.9 ± 1.8	12.3 ± 1.7	19.5 ± 2.4	< 0.0001	Groups II &IV vs. Groups II & III
Increase in MASE_S at stress echo (Mean ± SD)	7.0 ± 2.3	3.3 ± 2.0	3.1 ± 2.0	6.4 ± 2.7	< 0.0001	Groups II & IV vs. Groups II & III

Abbreviation:MASE, mitral annulus systolic excursion

## 4. Discussion

Prosthetic annuloplasty for the repair of mitral valve insufficiency was introduced by Carpentier and associates(30) . Long-term durability of repair is normally ensured by annular remodeling and fixation as the crucial end points of the surgical technique. There have been a host of studies into the use of prosthetic and biological devices in annuloplasty to date, yet there is still no consensus of opinion on the ideal annuloplasty ring.

Rings were originally introduced for the following purposes: 1) to accomplish the remodeling of annular deformity secondary to chronic atrial and ventricular enlargement, 2) to stabilize mitral repair by reducing the tension on reconstructed valvular portions, 3) to enhance leaflet coaptation, and 4) to prevent further annular dilatation.

The normal mechanics of the mitral valve apparatus require a dynamic annulus structure, especially on the posterior side. A rigid prosthetic fixation of the mitral annulus may reduce such a dynamic annular motion, affect the transmitral blood flow at diastole, and impair the LV function(15, 16, 21) , hence the support for flexible prosthetic devices to avert these complications(7, 8, 31) . Cooley and Bex proposed isolated posterior annuloplasty in mitral repair based on the anatomic concept that the mural portion of the mitral annulus is the only segment prone to dilation.(32) Many other investigators have since suggested other types of prosthetic supports to achieve selective annuloplasty(9-11) . Autologous pericardium rings in mitral repair were first utilized by Carpentier (33) , and pre-treated pericardial strips for posterior annular remodeling were an initiative of Salati’s(24) . We, however, sought to evaluate the use of untreated pericardial rings.

A number of studies have hitherto been carried out to compare the effects of rigid and flexible prosthetic rings on annular dynamics and LV performance after mitral repair. David et al(15) , having demonstrated that the short-term (2-3 months) benefits of flexible annuloplasty in terms of LV function were offset in a more limited number of patients at later follow-ups (2 years).They concluded that compensatory mechanisms of LV performance or impairment of ring flexibility could occur in the long term. Okada et al. arrived at the same results in 26 non-randomized patients, who underwent annuloplasty with the Carpentier or Duran flexible ring(21) . They showed that there was a better transmitral blood flow in patients with flexible annuloplasty and that the dynamic changes of the mitral surface area disappeared after the use of rigid annuloplasty rings. Rijk-Zwikker achieved unrestrictive annular remodeling with flexible devices, ensuring the maintenance of the physiological change of the mitral surface area(16) . Having made an intraoperative assessment of 12 patients undergoing Cosgrove-ring implantation along the mural annulus, Dall’Agata (22) reported that a 3-dimensional echocardiographic reconstruction of the mitral valve was indicative of preserved valvular mechanics early after the implantation of flexible C-shape devices. However, they did not observe any annular motion throughout the cardiac cycle after complete annular fixation with a rigid ring. Finally Chang et al. in their RCT enrolling 356 patients (Carpentier semi-rigid ring group, n=186; Duran flexible ring group, n=170), with similar demographics, showed similar long-term outcomes as well as left ventricular systolic function measured with echocardiography for the two groups at a mean follow-up of 46.6 months. The 8-year freedom from recurrence of significant MR was 62.6±19.0% in the Carpentier ring group and 55.5±14.1% in the Duran ring group(11).

All the above-mentioned investigations were comparative studies into the use of rigid and flexible prosthetic rings; nevertheless, the existing literature seems to be lacking in studies on pericardial annuloplasty. The first comparison between autologous pericardial rings and rigid prosthetic rings was performed by Borghetti et al.(27), who demonstrated that rigid prosthetic support exerted a constraining effect on annular dynamics, particularly in terms of diastolic motion. Pliable biological fixation of the mitral annulus in their patients appeared to favor the diastolic blood flow across the mitral valve at peak stress, suggesting enhanced annular motion and more effective valve-orifice area at diastole under demanding mechanical conditions. It is deserving of note that Borghetti’s investigation was performed on a highly selected non-randomized group of patients.

In the Bevilacqua study, there was a five-year freedom from reoperation in the pericardial ring group, and recurrent mitral regurgitation was significantly higher in patients with prosthetic rings than that in patients with pericardial rings (34).

Salati introduced pericardial rings and achieved not only good physiological mitral annular dynamics and ventricular performance during stress by comparison with rigid prosthetic rings, but also a 5-year freedom from reoperation of 89.7%(24) . In a retrospective study, Gillinov et al, showed that the type of annuloplasty (prosthetic or pericardial rings) had no impact on the long-term durability of repair. Nonetheless, they added that the Cleveland group ,over the last few years, has favored prosthetic ring annuloplasty over posterior pericardial annuloplasty(2). Bevilacqua in a non-randomized retrospective study identified pericardial annuloplasty as an independent risk factor for MVR failure and reoperation. Folliguet et al. reported that use of the pericardial valve results in a low incidence of valve-related complications such as leaflet calcification or structural deterioration(35) .

In our prospective randomized study on a larger and less specific population, we utilized biological material and witnessed the superiority of the pericardial annuloplasty concept in enhancing long-term LV function. We demonstrated that LV function, although not different between pericardial annuloplasty patients and normal cases, maintained a significant functional reserve during stress in patients with posterior pericardial annuloplasty. While we witnessed preserved and effective pericardial flexibility at late follow-up, we saw no gradual rigidity of the pericardial ring in the long term contrary to previously reported results of larger clinical series(25) , nor did we have any evidence of tissue calcification for up to 2-3 years after surgery.

In conclusion, posterior annular support with untreated autologous pericardium rings appears to be a good choice for the annuloplasty procedure, for it yields enhanced postoperative diastolic and systolic LV function during stress conditions as well as excellent annular dynamics without long-term degeneration.

Further comprehensive studies are required to evaluate autologous pericardium rings in terms of long-term durability and function of mitral valve repair and the postoperative LV function at rest and during stress conditions. Unless there are significant complications, totally biological valve reconstruction should be a safe option.
